# Filing the Teeth

**Published:** 1844-06

**Authors:** 


*Filing the Teeth.*—Mr. Robinson, in an article on filing the teeth, published in the *Forceps*, says, the teeth that are more generally attacked with caries, and for which the application of the file is more frequently brought into request, are the four central incisors and canines of the upper jaw, although in many instances it may be used with success to the bicuspides and molares of both jaws. The permanent central and lateral incisors of the upper jaw frequently decay at an early period at their sides. This arises either from a too crowded state of the mouth, and the undue influence exercised on the parts by their too rapid advance before the maxillary arch is sufficiently developed to admit the increased size, or from the patient at that period neglecting to perform those daily ablutions so essential and necessary to the health of these organs. In either case it unquestionably forms the exciting cause of caries in those situations, which, if allowed to extend beyond a certain point, renders the operation both difficult and dangerous to the tooth itself, owing to the confined space the operator has to use his instruments, with that force so requisite to the well packing of the gold to the exclusion of all foreign substances, with the liability of fracturing the enamel. Even if this difficulty should be overcome, the tooth may be broken in the attempt at stopping it, or the gold may become loose at the end of a few months. Hence arises the necessity of filing in the early stages of caries, in preference to stopping. In every stage which requires the use of the file, the dentist ought not to be content with merely dividing the teeth, but should extend the operation until the whole disease in the tooth is eradicated, and presents a surface as white as the healthy part of the tooth. A considerable portion of a tooth can be filed away without the slightest injury, if the operation be performed with caution, and the posterior portion removed without any perceptible disfigurement; in many cases, the caries can be removed by scraping away with an instrument, without having recourse to the file. Mr. Robinson has frequently, after dividing a tooth, discovered near its cutting edge a large cavity, which it would be impossible to remove without destroying more than half the tooth, and disfiguring the patient; in any attempt to stop it with gold, the chances would be, either a fracture or an imperfect stopping. In these cases, he has substituted gum-mastic steeped in water—an admirable substitute—which has remained in the cavity for months, and can be renewed at pleasure by the patient. In many instances, when the cavities have been examined three or four years afterwards, they have been found perfectly healthy, not in the least indicating a return of the disease.—*London Medical Times.—Boston Med. & Surg. Jour.*

### Miscellaneous Notices.

